# Multi-level remodelling of chromatin underlying activation of human T cells

**DOI:** 10.1038/s41598-020-80165-9

**Published:** 2021-01-12

**Authors:** Naiara G. Bediaga, Hannah D. Coughlan, Timothy M. Johanson, Alexandra L. Garnham, Gaetano Naselli, Jan Schröder, Liam G. Fearnley, Esther Bandala-Sanchez, Rhys S. Allan, Gordon K. Smyth, Leonard C. Harrison

**Affiliations:** 1 The Walter and Eliza Hall Institute of Medical Research, Parkville, 3052 Australia; 2 Department of Medical Biology, The University of Melbourne, Parkville, 3010 Australia; 3 School of Mathematics and Statistics, The University of Melbourne, Parkville, 3010 Australia

**Keywords:** Computational biology and bioinformatics, Genetics, Immunology

## Abstract

Remodelling of chromatin architecture is known to regulate gene expression and has been well characterized in cell lineage development but less so in response to cell perturbation. Activation of T cells, which triggers extensive changes in transcriptional programs, serves as an instructive model to elucidate how changes in chromatin architecture orchestrate gene expression in response to cell perturbation. To characterize coordinate changes at different levels of chromatin architecture, we analyzed chromatin accessibility, chromosome conformation and gene expression in activated human T cells. T cell activation was characterized by widespread changes in chromatin accessibility and interactions that were shared between activated CD4^+^ and CD8^+^ T cells, and with the formation of active regulatory regions associated with transcription factors relevant to T cell biology. Chromatin interactions that increased and decreased were coupled, respectively, with up- and down-regulation of corresponding target genes. Furthermore, activation was associated with disruption of long-range chromatin interactions and with partitioning of topologically associating domains (TADs) and remodelling of their TAD boundaries. Newly formed/strengthened TAD boundaries were associated with higher nucleosome occupancy and lower accessibility, linking changes in lower and higher order chromatin architecture. T cell activation exemplifies coordinate multi-level remodelling of chromatin underlying gene transcription.

Mammalian genomes are folded into highly organized hierarchical structures linked to function at each level^1^. At the primary level of chromatin structure, the nucleosome, a 147 base-pair DNA segment wrapped around an octamer of histone proteins, directly influences gene expression by dictating access of DNA to the transcriptional machinery^2–4^. At an intermediate level, the genome is organized into protein-mediated loops that facilitate 3-dimensonal (3D) interactions between of pairs of genomic sites such as promoters and enhancers distant within the genome. At a higher level, the genome is organized into self-interacting chromatin, called topologically associating domains (TADs)^5,6^. Disruption of TAD boundaries has been associated with developmental defects^7^ but the functional significance of changes in TAD architecture are otherwise largely unknown. Further, chromosomes are organized into a gene-rich, transcriptionally active compartment (A) with open chromatin and active histone marks and a gene-poor, transcriptionally inactive compartment (B) with condensed chromatin and gene silencing histone marks. Within this overall organization, the interplay between chromatin structure^8^ and gene expression^9–11^ is cell-specific and mediated by transcription factors (TFs) and other DNA binding proteins the functions of which depend on chromatin accessibility.

The immune system evolved to respond to environmental stimuli and exhibits a high degree of phenotypic and functional plasticity in response to external cues. T cells have a central role in the adaptive immune system and are activated under different conditions to expand and differentiate into a variety of specialized and functionally distinct subsets. T cell activation is likely to be instructive of how coordinate changes in chromatin structure orchestrate gene expression programs that underlie rapid and often lifesaving responses. It was recently shown that T cell activation is associated with a marked remodelling of chromatin structure at the level of accessibility (measured by ATAC-seq)^12–14^ and, separately, of conformation (measured by promoter capture-Hi-C)^15^. Here we analyse genome-wide chromosome conformation in conjunction with chromatin accessibility and whole transcriptome expression to further understand how coordinated changes across different levels of chromatin structure are linked to gene expression in response to T cell activation.

## Results

### Chromatin accessibility and 3D genomic interactions are immune cell-type specific

Resting CD4^+^ and CD8^+^ T cells, and mature B cells, were isolated from two healthy human male donors. T cells were activated through the T cell receptor subunit CD3 and the co-stimulator CD28 by incubation with anti-CD3/CD28 antibody Dynabeads for 72 h. Each cell lineage was profiled by assay for transposase-accessible chromatin using sequencing (ATAC-seq) to generate genome-wide maps of chromatin accessibility, by in situ Hi-C for 3D genomic interactions and by RNA-seq for whole transcriptome expression. The consistent use of two independent biological replicates for each cell population across all three genomic technologies allowed us to assess activation-induced changes in an integrated and statistically rigorous way. For each technology, a set of relevant genomic features was identified and held fixed across each cell populations. For ATAC-seq, the features were identified peaks, for Hi-C the features were pairs of genomic windows and for RNA-seq the features were genes. Activation-induced changes were explored by comparing activated to resting T cells and the statistical significance of the changes was determined with the edgeR^16^ and limma^17^ software packages, controlling the false discovery rate relative to biological variability between the individual donors.

**Figure 1:**
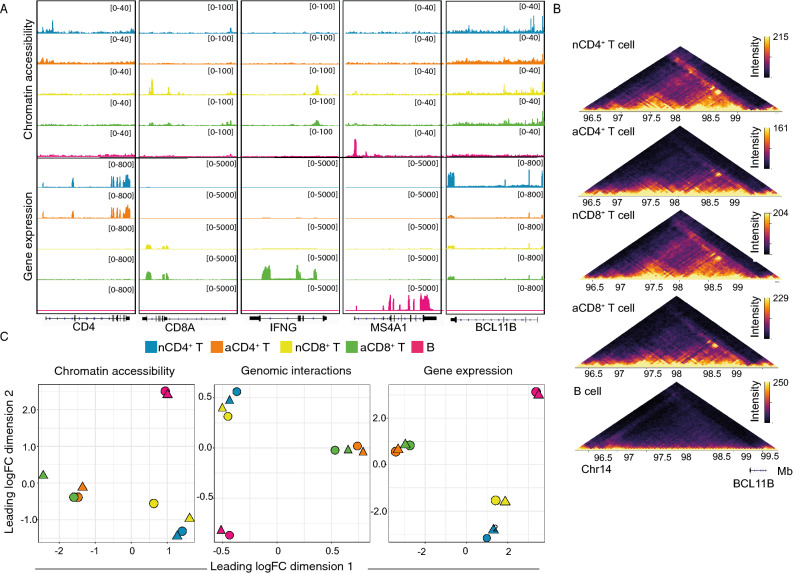
Chromatin structure and gene expression are immune cell type-specific. (**A**) Normalized read coverage plots of ATAC-seq and RNA-seq libraries at phenotype-defining genes including *CD4*, *CD8A, IFNG, MS4A1* and *BCL11B* loci in resting CD4^+^ (nCD4^+^), resting CD8^+^ (nCD8^+^), activated CD4^+^ (aCD4^+^), activated CD8^+^ (aCD8^+^) T and resting B (**B**) cells. (**B**) In-situ Hi-C contact matrices for a 1 Mb region on chromosome 14 that includes BCL11B, which is known to be expressed in T but not B cells. The top four Hi-C matrices display data from resting and activated T cells and show distinct genome organisation at the gene, while the bottom Hi-C matrix of B cells shows a lack of genome organisation at BCL11B. Color scale indicates number of reads per bin pair and has been scaled within each matrix to facilitate comparisons. (**C**) Multidimenional scaling (MDS) plots of log-CPM values with samples coloured by cell type and shaped by donors. The log-CPM values were corrected for the donor variable. Distances on the plot correspond to the leading fold-change, which is the average (root-mean-square) log2-fold-change for the 500 genes (RNA-seq), 5000 peaks (ATAC-seq) and 50,000), bin pairs (Hi-C) most divergent between each pair of samples.

First, we confirmed the reproducibility and specificity of the data. ATAC-seq, Hi-C-seq and RNA-seq sequencing read densities at genes that define the cell phenotypes confirmed specificity for each cell type (Fig. 1A–C). We observed a strong enrichment of ATAC-seq reads at transcription start sites (TSSs) genome-wide, including promoter-TSSs of the cell type-specific genes *CD4, CD8A, IFNG, BCL11B and MS4A1,* which reflected the quality of the data^18^ (Fig. 1A,B). T cells, but not B cells, exhibited robust enrichment of Hi-C genomic interactions at the BCL11B gene, known to be expressed in T but not B cells^19^ (Fig. 1C). Conversely, B cells exhibited robust enrichment of Hi-C genomic interactions at the BCL11A gene, known to be expressed in B but not T cells (Supplementary Fig. S1). Unsupervised clustering of the samples by multidimensional scaling of the chromatin accessibility, chromatin interaction and gene expression data demonstrated distinct chromatin structure and gene expression signatures for the different cell types (Fig. 1D), as previously reported for chromatin architecture^8^. Samples clustered by activation status over dimension 1 and diverged by cell lineage over dimension 2. By calculating the proportion of variation explained by each dimension using the Glimma package (v1.6.0)^20^, activation (dimension 1) was found to account for the largest proportion of variation in chromatin accessibility, chromatin interactions and gene expression (34%, 27% and 49%, respectively), followed by lineage (dimension 2) (23%, 19% and 17%) (Fig. 1D). Consistent with the smaller contribution of T cell lineage to biological variance in chromatin accessibility and interactions, direct comparison of activated CD4^+^ and CD8^+^ T cells revealed only a small number of chromatin structure differences, viz. 864 differentially accessible regions and 69 differential interactions, indicating that chromatin remodelling in response to activation is similar in CD4^+^ and CD8^+^ T cells.

### T cell activation leads to extensive changes in chromatin accessibility and gene expression

**Figure 2:**
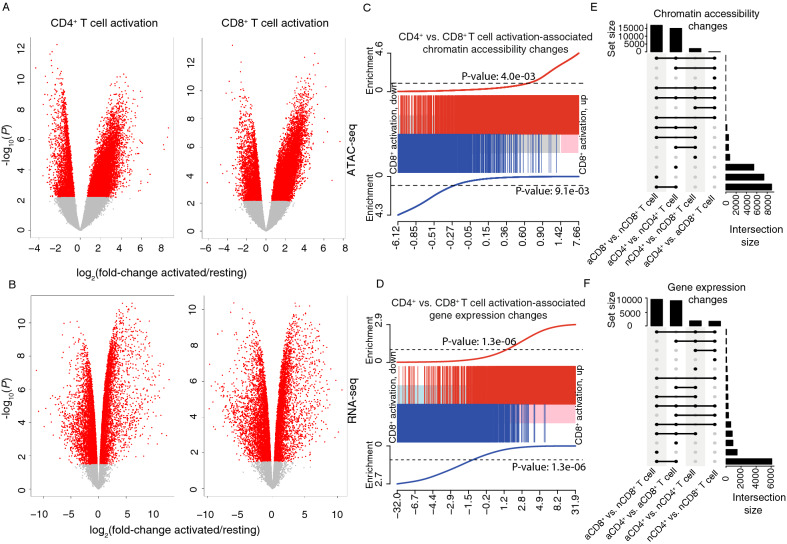
T cell activation induces extensive changes in chromatin accessibility and gene expression that are comparable between CD4^+^ and CD8^+^ T cells. (**A**) Volcano plots showing the magnitude of differential accessibility in activated CD4^+^ and CD8^+^ T cells. Each point represents a peak with significant changes highlighted in red (FDR < 0.05). (**B**) Volcano plots showing the magnitude of differential expression in activated CD4^+^ and CD8^+^ T cells. Each point represents a gene with significant changes in red (FDR < 0.p05). (**C**) Barcode enrichment plot showing that DA peaks in CD4^+^ T cells are similarly ranked in CD8^+^ T cells. Peaks are ordered from left to right on the plot from most down-regulated to most up-regulated upon activation in CD8^+^ T cells, forming a shaded middle horizontal bar, with log2-fold-changes shown by the x-axis. DA peaks significantly up-or down-regulated upon activation in CD8^+^ T cells (FDR < 0.05) are marked by red and blue vertical bars, respectively. The red enrichment worm at the top of the plot shows the local density of red vertical bars while the blue enrichment worm at the bottom of the plot shows the local density for blue vertical bars. P-values show significance of the enrichments (fry gene set tests). The enrichment pattern shows strong concordance of the CD4^+^ and CD8^+^ changes. (**D**) Barcode enrichment plot showing that DE genes in CD4^+^ T cells are similarly ranked in CD8^+^ T cells. (**E**) UpSet plot showing the number of DA peaks for each comparison (activated vs. resting CD8^+^ T cells, activated vs. resting CD4^+^ T cells, resting CD4^+^ vs. resting CD8^+^ T cells and activated CD4^+^ vs. activated CD8^+^ T cells) and their intersections. Vertical bars illustrate the total number of DA peaks for each comparison. Horizontal bars show the number of common DA peaks (intersection size) for a given set of comparisons (filled connected circles). (**F**) UpSet plot showing the number of DE genes for the same comparisons as in (**E**).

Relative to the resting state, T cell activation increased accessibility of 11,368 (10.0%) and 12,282 (10.8%) ATAC-peaks in CD4^+^ and CD8^+^ T cells, respectively, while 3674 (3.2%) and 4,723 (4.1%) peaks lost accessibility (Fig. 2A, Supplementary Table S1). Activation altered a higher proportion of expressed genes, with 4894 (33.4%) and 4727 (32.3%) being up-regulated and 4931 (33.7%) and 4611 (31.5%) in CD4^+^ and CD8^+^ T cells, respectively (Fig. 2B). Interestingly, despite differences in their effector functions, activation-associated changes in chromatin accessibility and gene expression were highly concordant in CD4^+^ and CD8^+^ T cells, evidenced by their strong correlation (Fig. 2C,D) and large number of overlapping activation-associated chromatin accessibility (9083) and gene expression (7823) changes (Fig. 2E,F; ‘aCD4^+^ vs. nCD4^+^ T cell’ and ‘aCD8^+^ vs. aCD8^+^ T cell’ comparisons). Thus, differentially accessible (DA) peaks and differentially expressed (DE) genes up-and down-regulated upon activation of CD4^+^ T cells were also up-and down-regulated, respectively, upon activation of CD8^+^ T cells (Fig. 2C,D). Accessibility and gene expression differences between activated CD4^+^ and CD8^+^ T cells were also minimal (Fig. 2E,F; ‘aCD4^+^ vs. aCD8^+^ T cell’ comparison). Moreover, in interrogating the DA peaks for enrichment of known transcription factor motifs we observed that activation-associated accessibility changes in both CD4^+^ and CD8^+^ T cells were enriched for a similar set of DNA motifs recognized by TFs involved in T cell development, activation or proliferation^10,21–25^ (Supplementary Fig. S2A). These included members of the bZIP (BATF, FOSL1, ATF3), ETS (Fli1, ETV1, ERG, GABPA), and Runt (RUNX1, RUNX2) families among others (Supplementary Fig. S2A). Comparison with ATAC-seq signatures (GSE118189) reported in human CD4^+^ and CD8^+^ T cells after 24 h activation^10,21–25^, revealed that 24 and 72 h activation-associated accessibility changes were also highly concordant (Supplementary Fig. S3A), had a large number of overlapping DA peaks (Supplementary Fig. S3B) and were enriched for a similar set of TF motifs (Supplementary Fig. S3C). Thus, both the earlier and later stages of CD4^+^ and CD8^+^ T cell activation would appear to be governed by similar chromatin regulatory and transcription factor programs.

Using annotations from the Refseq database and predicted regulatory states from ChromHMM in the Roadmap Epigenomics Project^26^, we annotated the activation-associated DA peaks and calculated their enrichment over the universe of ATAC-seq peaks using GAT^27^. Overall, approximately 77% of the activation-induced peaks were located in intronic and intergenic regions while the remaining were at promoters (defined from − 1 kb to + 100 bp of the TSS) (~ 14%) and exonic regions (~ 3%). Moreover, DA peaks showed strong enrichment for ChromHMM-predicted promoters and enhancer regions, and depletion in repressive marks (heterochromatin, quiescent DNA and repressed PolyComb) (Supplementary Fig. S2B).

Several studies have shown that disease-associated SNPs frequently reside in cell-specific regulatory elements^28–30^. We therefore tested whether regions of altered chromatin accessibility associated with T cell activation were enriched for disease-/trait-associated SNPs or those in linkage disequilibrium (LD) with them (Supplementary Table S2). After categorizing SNPs for 15 disease or trait classes, we observed a strong enrichment for immune system, neurological autoimmune, haematological and cancer SNPs in DA peaks compared to the universe of ATAC-seq peaks (Supplementary Fig. S2C). Moreover, enrichment was more pronounced in the subset of peaks that contained predicted enhancers by ChromHMM (Supplementary Fig. S2C).

Because whole transcriptome sequencing without poly(A) pre-selection allowed us to detect enhancer RNAs^15^, we could identify the subset of enhancer (e)RNAs that overlapped the set of human enhancer regions defined by the FANTOM consortium^31^. In order to investigate if variation in chromatin accessibility was related to changes in eRNA expression, and thus enhancer activity, we calculated the distribution of eRNA expression across the DA peaks. We observed that eRNA expression was on average significantly higher in the chromatin regions that gained accessibility upon activation than in those that lost accessibility (Supplementary Fig. S4A).

To further explore the relationship between the activation-associated DA peaks and expression of their potential target genes we applied the Genomic Regions Enrichment of Annotations Tool (GREAT)^32^. We observed that expression of genes linked to the peaks that gained accessibility was on average significantly higher than that of genes linked to peaks that lost accessibility (Supplementary Fig. S4B). The set of target genes defined by GREAT comprised immune system-associated genes known to be up-regulated upon T cell activation, including TNFSF15, TNFRSF8, IL32, IL23R, IL12RB2, IL1R2, IL2, IL21 and IL13 among others. Furthermore, functional enrichment analysis by GREAT showed that DA peaks were significantly enriched for a number of relevant Gene Ontology terms, including regulation of immune system process, regulation of response to stimulus or regulation of cellular processes, among others.

In summary, this integrated analysis reveals that T cell activation leads to extensive remodelling of chromatin accessibility and the formation of active regulatory chromatin regions associated with TFs relevant to T cell biology, enhancer activity and expression of genes critical in T cell responses, comparable between CD4^+^ and CD8^+^ T cells.

### T cell activation leads to genome-wide changes in chromatin interactions

**Figure 3:**
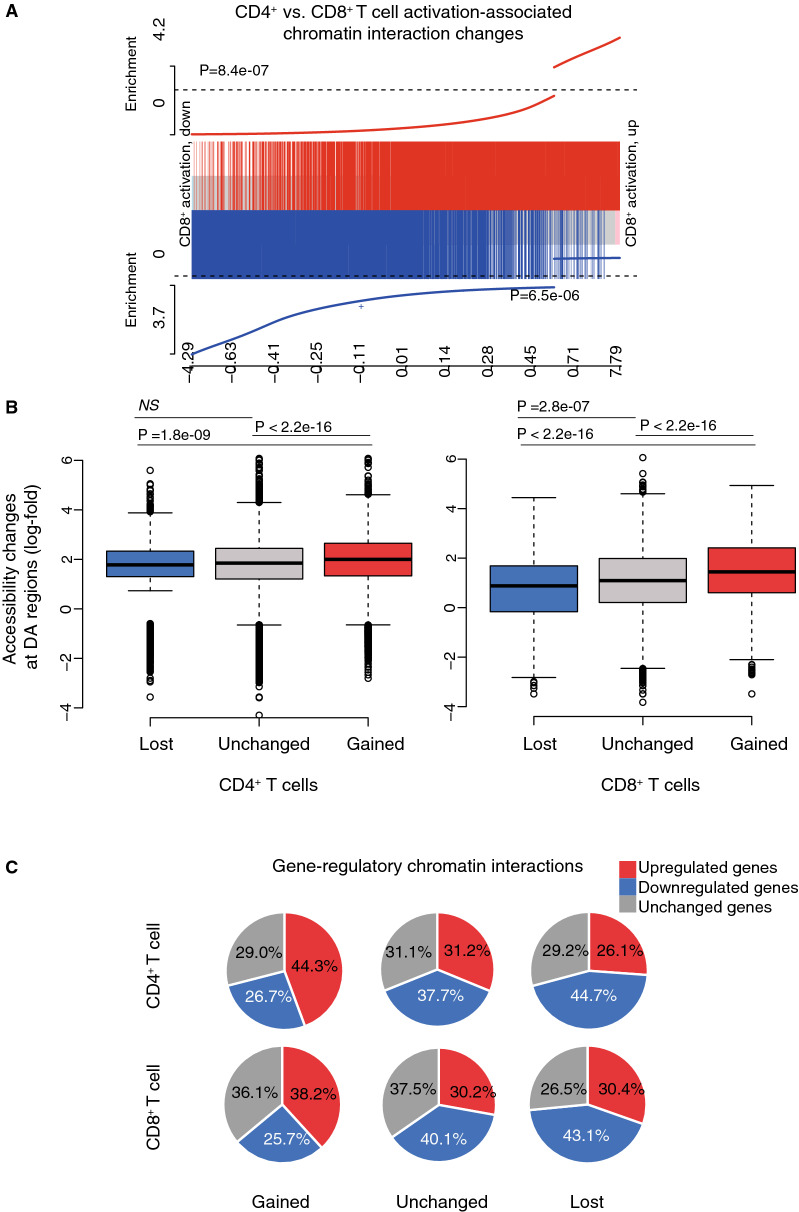
T cell activation induces genome-wide changes in gene-regulatory interactions. (**A**) Barcode enrichment plot showing that activation-associated DIs in CD4^+^ T cells are similarly ranked in CD8^+^ T cells. The plot is exactly analogous to Figs. 2C,D but for DI bin-pairs instead of DA peaks or DE genes. (**B**) Box plots showing that the increased accessibility at DA regions in gene-regulatory chromatin interactions ‘gained’ is higher than that at gene-regulatory chromatin interactions ‘unchanged’ or ‘lost’, following CD4^+^ (left) and CD8^+^ (right) T cell activation. Shown are the median (central horizontal line), interquartile range (boxes), values of the upper and lower quartiles (whiskers), outliers beyond 1.5 IQR (circles). Statistical comparisons were made with the unpaired Wilcoxon test. (**C**) Pie chart showing distribution of down-regulated, up-regulated or unchanged target genes linked to ‘lost’ (top) or ‘gained’ (bottom) of regulatory interactions..

Chromatin interactions bring disparate regulatory elements and genes into spatial proximity to regulate transcription. To identify how the chromatin interactions change upon T cell activation we used the diffHic pipeline^33^ in conjunction with edgeR. The pipeline partition the genome into 25 kbp bins. Each pair of bins from the same chromosome represents the location of a potential chromatin interaction, with the intensity of the interaction measured by the number of reads mapping to that bin-pair. Statistical tests for gain or loss of intensity were performed for each bin-pair. Upon activation, we found 17,965 and 15,496 interactions were gained in CD4^+^ and CD8^+^ T cells, respectively, and 12,434 and 11,376 interactions were lost (Supplementary Tables S3A and S3B). Across the whole genome, 103,741 bins had above-background interaction activity and more than half of these (56.3% in CD4^+^ T cells and 52.4% in CD8^+^ T cells) were involved in a least one significant differential interaction (DI). Similar to the chromatin accessibility and gene expression changes upon activation, activation-associated chromatin interaction changes were highly concordant between the CD4^+^ and CD8^+^ T cells, reflected by the strong positive correlation (Fig. 3A).

Examples of highly ranked DIs are shown in Supplementary Fig. S5. The figures show the interactions that are gained and lost on activation together with the associated genes that are DE and regions that are DA. We next identified a network of chromatin interactions connecting DA peaks with the promoters of expressed genes, which we refer to as gene-regulatory chromatin interactions. Regions with gene-regulatory chromatin interactions ‘gained’ were associated with a larger increase in chromatin accessibility compared to those with ‘lost’ or ‘unchanged’ interactions following activation of either CD4^+^ or CD8^+^ T cell (Fig. 3B). Furthermore, when we examined if ‘gained’ or ‘lost’ regulatory interactions were associated with up-regulation or down-regulation of gene expression we found that that the proportion of ‘gained’ gene-regulatory interactions associated with up-regulated genes was significantly higher than that associated with ‘lost’ or ‘unchanged’ gene-regulatory interactions (Fisher’s exact test p-value < 1.1 × e − 11), while the proportion of ‘lost’ gene-regulatory interactions associated with down-regulated genes was also significantly higher than those linked to ‘gained’ or ‘unchanged’ gene-regulatory interactions (Fisher’s exact test p-value < 5 × e − 10) (Fig. 3C). Thus, ‘gain’ and ‘loss’ interactions are coupled, respectively, to up-regulation and down-regulation of corresponding target genes.

### T cell activation is associated with partitioning of genome topology

**Figure 4:**
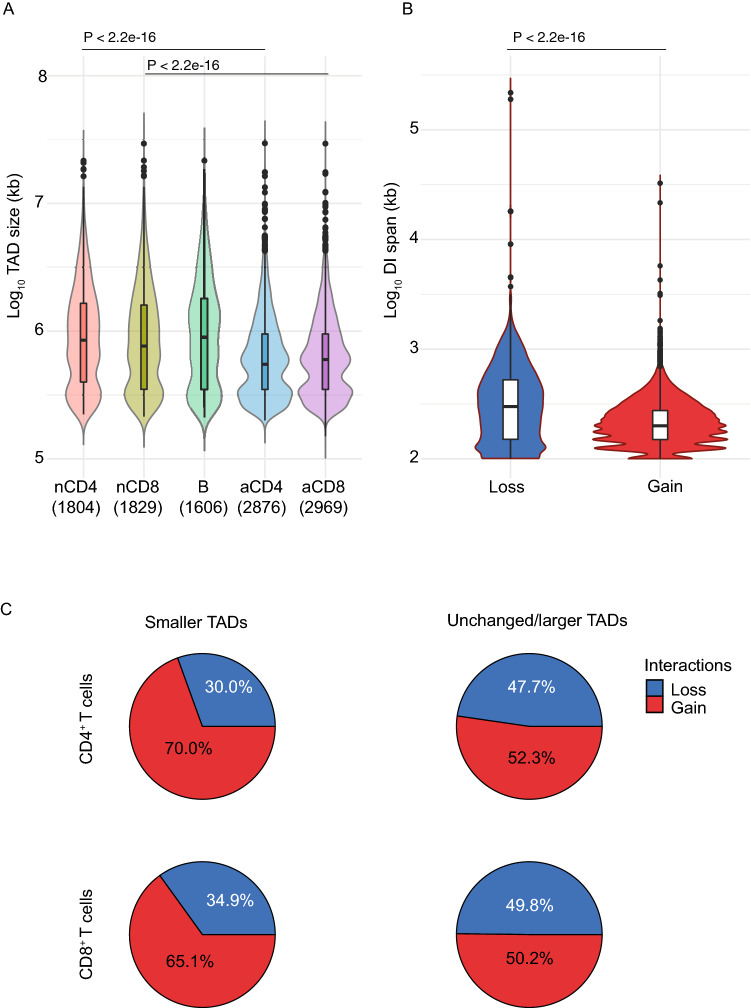
T cell activation results in partitioning of genome topology. (**A**) Violin plots showing the distribution of TAD sizes in resting and activated T cells and B cells determined from summed libraries called with TADbit. Numbers in parenthesis depict the number of TADs called in each category. (**B**) Violin plots showing the distribution of the span of differential chromatin interactions (DIs) determined at 25 kbp resolution (FDR < 0.05) that are lost (decrease in logFC) or gained (increase in logFC) upon activation. Violin plots show median (horizontal line), interquartile range (boxes), values of the upper and lower quartiles (whiskers), outliers (circles) and kernel density estimation. Data for CD4^+^ and CD8^+^ T cells were merged as they showed a similar pattern. (**C**) Pie charts showing distribution of Loss and Gain chromatin interactions in TADs that become smaller in size (left) or remain unchanged/became larger in size (right) upon T cell activation. Statistical comparisons were made with the unpaired Wilcoxon test.

Evidence has emerged of the dynamic nature of TAD and DNA loop formation, mainly from studies of mouse embryo development (reviewed in Ref.^1^). To examine TAD plasticity we first segmented the chromatin of resting and activated T cells, and B cells, into TADs using TADbit^34^. Naïve CD4^+^ T, CD8^+^ T and B cells had 1727, 1750 and 1602 TADs with a mean size of 1.40, 1.38 and 1.51 Mb, respectively; activated CD4^+^ and CD8^+^ T cells had 2772 and 2863 TADs with a mean size of 0.85 and 0.84 Mb, respectively (Fig. 4A). In activated T cells, TADs appeared to be partitioned, becoming smaller and more numerous than in resting T cells (Fig. 4A). We next explored the proportion of intersecting TADs among different TAD sets. TADs were termed intersecting if the reciprocal region overlap was higher than 75%. On average, 62% of the TADs intersected between resting CD4^+^ T and CD8^+^ T cells and 70% of TADs intersected between activated CD4^+^ T and CD8^+^ T cells. Interestingly, this overlap fell to an average of 43.5% when comparing resting and activated T cells. Further analysis of the TAD intersects revealed that while only 24% of the TADs called in resting CD4^+^ T cells overlapped two or more TADs in resting CD8^+^ T cells this increased to 44% when analysing the TAD overlap between resting and activated T cells (Supplementary Fig. S6). This suggests that T cell-activation induces partitioning of TADs into smaller chromatin domains.

**Figure 5:**
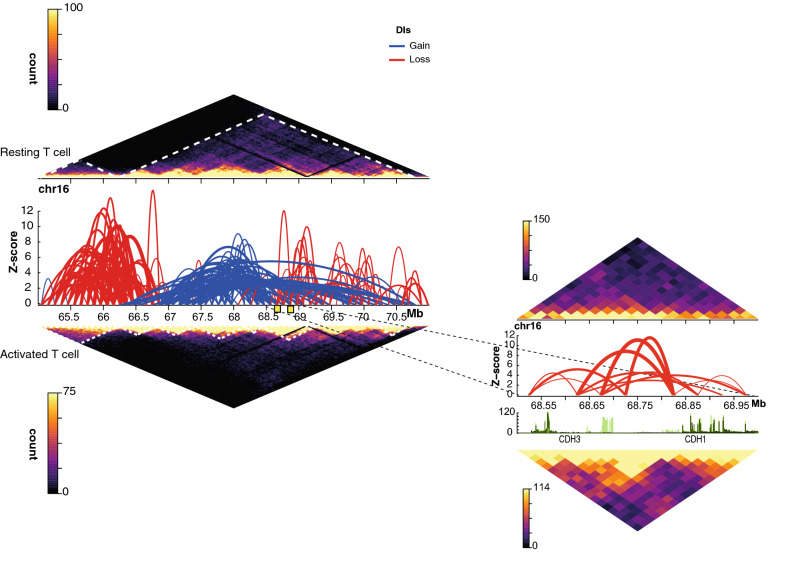
T cell activation results in partitioning of genome topology. In-situ Hi-C contact matrices plotted at 50 kbp resolution in resting and activated T cells over genes that have been associated with T cell activation such as CDH3 and CDH1. Colour scale of contact matrix indicates number of reads per bin pair. Locations of the TADs are marked by white dotted lines as called by TADbit. Significant differential interactions (DIs) as determined by the diffHic pipeline at 100 kbp (FDR < 0.05) are represented by arcs in the centre, where the vertical axis is the z-score (− log_10_ p-value as calculated by edgeR). Each arc connects the two interacting bins and therefore identifies the relevant bin pair. Red and blue arcs represent strengthened (logFC > 0) and weakened (logFC < 0) DIs, respectively, in response to activation. The zoomed inset regions show in-situ Hi-C contact matrices and DIs at 25 kbp resolution (FDR < 0.05) and RNA sequencing coverage plots at the CDH3 and CDH1 loci. Light and dark green coverage plots represent activated and resting T cells, respectively.

We observed also that, analogous to TAD partitioning, chromatin interactions that strengthened upon activation were on average significantly shorter than those that were weakened (t = 9.3; P-value < 2e−16), i.e. activation appeared to preferentially promote disruption of the longer-range interactions (Fig. 4B). Remarkably, intra-TAD chromatin interactions in TADs that became smaller upon activation showed a higher proportion of ‘gain’ interactions than those in TADs that remained unchanged or increased (Fig. 4C). This suggests that TADs have a pivotal role in shaping the intra-TAD chromatin loops. Examples of TAD partitioning and rearrangement of chromatin interactions are shown (Fig. 5 and Supplementary Fig. S7); significant differential interactions in response to activation are represented by red (‘gained’) and blue (‘lost’) arches, and locations of the TADs in resting (top) and activated (bottom) T cells are shown by yellow triangles.

We next sought to quantify how TADs changed upon T cell activation. Like most TAD caller algorithms, TADbit uses a binary approach whereby TAD boundaries are either present or completely absent, and thus TADbit is unable to identify subtle changes in genome architecture characterized by the quantitative strengthening or weakening of a boundary. Therefore, we used diffHic and edgeR to test for strengthening or weakening of each of the TAD boundaries. Of the 7260 and 7337 unique TAD boundaries called by TADbit, 3529 (46.0%) and 3755 (51.2%) were called differential by edgeR. Of these, 1596 and 1440 became significantly stronger and 1933 and 2315 weaker in CD4^+^ and CD8^+^ T cells, respectively. Thus, our findings indicate that T cell activation elicits a significant remodelling of the TAD boundaries.

**Figure 6:**
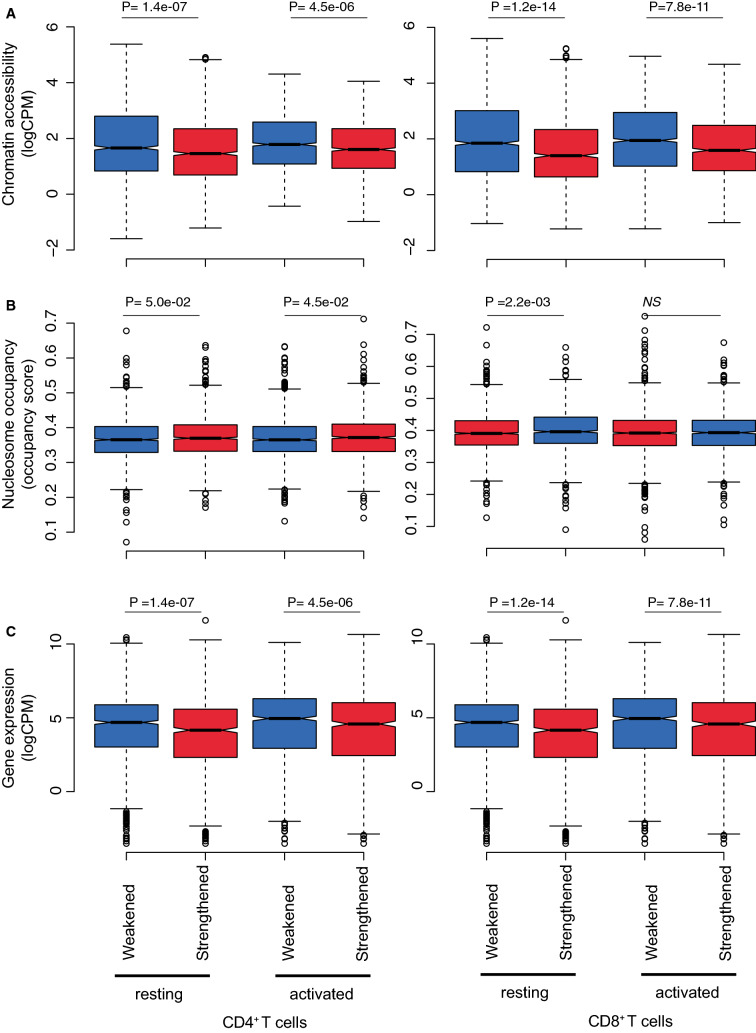
Increased strength in TAD boundary and chromatin interactions upon T cell activation is associated with nucleosome occupancy and gene expression. Box plots showing the distribution of the average (**A**) chromatin accessibility (logCPM), (**B**) nucleosome occupancy (NucleoATAC occupancy score) and (**C**) expression of protein-coding genes (logCPM) across differential TAD boundaries strengthened and weakened following activation of CD4^+^ and CD8^+^ T cells. Shown are the median (central horizontal line), interquartile range (boxes), values of the upper and lower quartiles (whiskers), outliers beyond 1.5 IQR (circles). Statistical comparisons were made with unpaired Wilcoxon tests.

We then calculated the distribution of chromatin accessibility, nucleosome occupancy and gene expression for the differential TAD boundaries in both resting and activated T cells. Interestingly, we observed that chromatin regions overlapping TAD boundaries that strengthened upon activation were associated on average with lower chromatin accessibility, higher nucleosome occupancy and lower protein-coding gene expression than those overlapping TAD boundaries weakened (Fig. 6). This trend was true for both resting and activated T cells, implying that chromatin regions associated with TAD boundary formation/disruption in T cells have intrinsic invariant features. In summary, T cell activation leads to global rearrangement of TAD boundaries, partitioning of TADs and disruption of long-range chromatin interactions, with weakening of TAD boundaries associated with regions of higher accessibility and gene expression.

### Chromosome A/B compartments are not altered substantially by T cell activation

**Figure 7:**
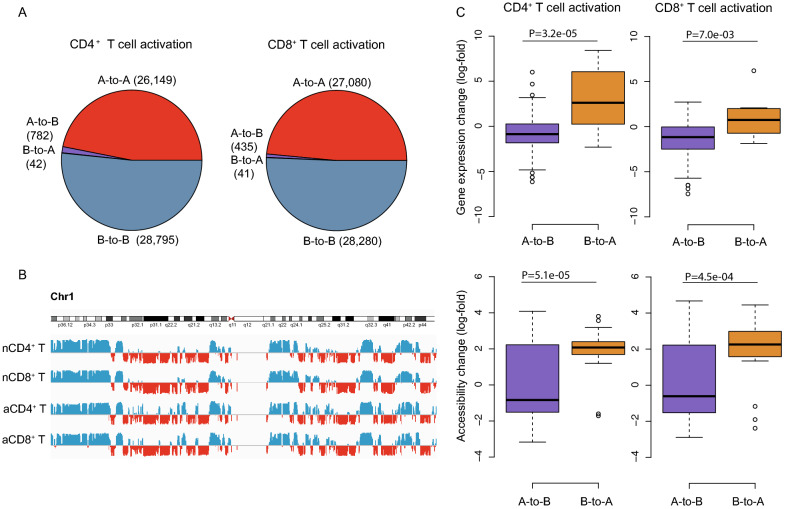
Chromosome A/B compartments are not substantially altered by T cell activation. (**A**) Pie charts showing the distribution of chromatin regions in the four different group of compartment changes: stable (A to A, B to B) or flipping (A to B and B to A). This was defined by pairwise comparison between activated and resting T cells. (**B**) PCA (principal component analysis) scores in resting and activated T cells along chromosome 1 representing the stability of the A/B compartments. PCA scores were calculated as described in “Methods”. (**C**) Boxplots showing the distribution of chromatin accessibility and gene expression at regions that exhibit A/B compartment shifts upon T cell activation. Shown are the median (central horizontal line), interquartile range (boxes), values of the upper and lower quartiles (whiskers), outliers beyond 1.5 IQR (circles). Statistics were determined with the unpaired Wilcoxon test.

Lineage differentiation is associated with rearrangement of chromosome A/B compartments^35^. However, in response to T cell activation we observed only modest changes in the A/B compartments, with less than 824 (1.4%) and 476 (0.8%) of the genome switching compartments upon activation CD4^+^ and CD8^+^ T cells, respectively (Fig. 7A,B). As expected, the small proportion of regions that switched from A to B showed decreased accessibility and gene expression, whereas regions that switched from B to A tended to show higher accessibility and gene expression (Fig. 7C). Changes across T and B cell types were also minimal. Overall, the pattern of change was subtle, indicating that T cell activation has minimal impact on the A and B compartments.

## Discussion

Previous studies have characterized either activation-responsive chromatin accessibility^13,14,36,37^ or 3D organization of the genome^8,13,15^ in mouse and human immune cells. However, to our knowledge no previous study has characterized chromatin accessibility, Hi-C chromatin interactions and the whole transcriptome in concert in response to activation of human CD4^+^ and CD8^+^ T cells. By using biological replicates throughout, changes in chromatin accessibility, interaction intensity and gene expression could be rigorously tested statistically, relative to the biological variation observed between human individuals. Activation of T cells was associated with multi-level remodelling of chromatin that was comparable between CD4^+^ and CD8^+^ T cells, indicating that their activation is regulated by similar genome control mechanisms. In response to T cell activation, we observed extensive changes in chromosome accessibility in regions enriched for regulatory elements and eRNAs, immune system-associated SNPs and TFBSs recognized by TFs involved in T cell development, activation or proliferation. These findings parallel those in other systems^12–14,36,37^ and are consistent with the view that changes in chromatin accessibility in response to external cues identify active regulatory regions likely to be critical for transcriptional responses. Furthermore, we also show that T cell activation uncovers many additional regulatory regions likely to be associated with the immune response and induces thousands of differential chromatin interactions linked to up-regulation or down-regulation of the corresponding target genes. At a higher level of chromosome architecture, T cell activation was associated with genome-wide rearrangement of TAD boundaries.

Although TADs were reported to be largely invariant across species and cell types^5,6^ our findings indicate otherwise and, in accord with recent evidence^1^, reveal that TADs are dynamic. We found that T cell activation was associated with global rearrangement of chromatin interactions involving changes in the strength of TAD boundaries and a smaller compartment structure. Strengthened TAD boundaries displayed lower chromatin accessibility, higher nucleosome occupancy and lower gene expression both before and after activation. Nucleosome organization is known to dictate local chromatin folding by regulating internal factors including linker DNA length and linker-histone binding affinities, but whether nucleosome architecture influences higher level genome topology, i.e. TADs and DNA loops, remains unknown^38^. Polymer simulations of the chromatin fiber suggest that regions of “stiffness” can act as local insulators to decrease interaction frequencies^39^. In mammary epithelial MCF-10A cells, knockdown of BRG1, the major ATPase subunit of the SWI/SNF chromatin remodelling complex, globally altered long-range genomic interactions and decreased TAD boundary strength, suggesting that nucleosome occupancy around CTCF sites might contribute to the regulation of higher-order chromatin architecture^40^. Our results in primary human cells support the view that long-range chromosomal organization and nucleosome occupancy are molecularly coupled and suggest that chromatin regions associated with TAD boundary formation have invariant features reflected by lower accessibility and higher nucleosome occupancy in both resting and activated T cells. Further examination of other elements at differential TAD boundaries may provide valuable insights into the TAD formation process and its role in chromatin folding and transcriptional regulation.

Together, our findings connect multi-level features of chromatin structure to gene expression in response to T cell activation. In addition, we provide a genome-wide resource of T cell activation-associated regulatory regions that will aid interpretation of chromatin accessibility data in human T cells.

## Methods

### Blood collection, cell subset isolation and T cell activation

Heparinised venous blood was collected at 09.00 h from two healthy young adult males, who gave written informed consent. The research was carried out in accordance with the principles of the Declaration of Helsinki and the guidelines of Nature journals and was approved by the Walter and Eliza Hall Institute of Medical Research Human Research Ethics Committee (application 88/03). Peripheral blood mononuclear cells (PBMCs) were purified by Ficoll-Hypaque gradient centrifugation and cryopreserved in liquid N2. Thawed PBMCs, ≥ 92% viable by acridine orange-ethidium bromide staining, were stained with anti-human αβ TCR (eBioscience, clone IP26, cat. no. 46-9986-42), anti-human CD4 (BD Pharmingen, clone RPA-T4, cat. no. 555349), anti-human CD45RA (BD Pharmingen, clone 5H9, cat. no. 556626), anti-human CD25 (BD Pharmingen, clone M-A251, cat. no. 557741), anti-human CD14 (BioLegend, clone 63D3, cat. no. 367104), anti-human CD16 (BD Pharmingen, clone 3G8, cat. no. 557758), anti-human HLA-DR (eBioscience, clone L243, cat. no. 48-9952-42), and anti-human CD19 (BioLegend, clone HIB19, cat. no. 302238). Naïve CD4^+^ T cells (CD14^−^ CD16^−^ TCRαβ^+^ CD4^+^ CD45RA^+^CD25^−^) and CD8^+^ T cells (CD14^−^CD16^−^TCRαβ^+^CD4 + ^-^CD45RA^+^CD25^-^), and B cells (TCRαβ^-^, HLA-DR^+^, CD19^+^), were flow-sorted on a FACSAria (BD Biosciences). Resting CD4^+^ and CD8^+^ T cells were cultured in Iscove’s Modified Dulbecco’s Medium containing 5% pooled, heat-inactivated, human serum, 100 nM non-essential aminoacids, 2 mM of glutamine and 50 μM 2-mercaptoethanol (IP5) medium. T cells were activated through the T cell receptor subunit CD3 and the co-stimulator CD28 by the addition of Human T-Activator CD3/CD28 Dynabeads at a 1:2 bead:cell ratio (Life Technologies, cat. no. 111.31D) for 72 h.

### RNA-seq

RNA was isolated using the miRNeasy Micro Kit (QIAGEN, cat. no. 217084). RNA libraries were prepared with an Illumina's TruSeq Total Stranded RNA kit with Ribo-zero Gold (Cat# RS-122-2001, Illumina) according to the manufacturer’s protocol. rRNA-depleted RNA was purified and then reverse transcribed into cDNA using SuperScript II reverse transcriptase (Cat# 18064014, Invitrogen). Validation of the library preparations was performed using Agilent D1000 ScreenTape Assay on the Agilent 2200 TapeStation. Total RNA-Seq libraries were sequenced on the Illumina NextSeq 500 platform to produce 2 × 75 paired-end reads. All samples were aligned to the human genome, build hg38, using the Rsubead aligner v1.24.1^41^. The number of fragments overlapping each Entrez gene were summarised using featureCounts^42^ and Rsubread's inbuilt hg38 annotation. Gene symbols were associated with Gene IDs using NCBI gene information (ftp://ftp.ncbi.nlm.nih.gov/gene/DATA/GENE_INFO).

### ATAC-seq

ATAC-seq was performed as previously described^18^. For each immune cell subset, 50,000 purified cells were lysed in cold lysis buffer (10 mM Tris–HCl, pH 7.4, 10 mM NaCl, 3 mM MgCl_2_ and 0.03% Tween20). Immediately after lysis, nuclei were spun out at 500 g for 8 min at 4 °C and the supernatant carefully removed. Nuclei were resuspended with Tn5 transposase reaction mix (25 µl 2X TD buffer, 2.5 µl Tn5 transposase, and 22.5 µl nuclease- free water) (Nextera DNA Library Prep Kit (Illumina, cat. no. FC-121-1030). The transposase reaction was performed at 37 °C for 30 min, and DNA immediately purified with a Qiagen MinElute kit (QIAGEN, cat. no. 28204). ATAC-seq libraries were sequenced on an Illumina NextSeq 500 to produce 75 bp paired-end reads.

### In situ Hi-C

In situ HiC was performed as previously published^43^. Primary immune cell libraries were generated in biological duplicates. Libraries were sequenced on an Illumina NextSeq 500 to produce 81 bp paired end reads. Between 129 and 280 million valid read pairs were generated per sample.

### Differential gene expression analysis

Differential expression analyses were undertaken using the edgeR v3.20.9^44^ and limma v3.34.9^17^ software packages. Any gene which did not achieve a count per million mapped reads (CPM) greater than 1.5 in at least 2 samples were deemed to be unexpressed and subsequently filtered from the analysis. Additionally, all genes without current annotation were also removed. Compositional differences between libraries were normalized using the trimmed mean of log expression ratios method (TMM)^45^. All counts were then transformed to log2-CPM with associated precision weights using voom^46^. Differential expression between all cell types was assessed using linear models and robust empirical Bayes moderated t-statistics^47^. To increase precision, the linear models incorporated a correction for a donor batch effect. P-values were adjusted to control the FDR below 5% using the Benjamini and Hochberg method.

### eRNA analysis

Reads overlapping the non-exonic ATAC-seq peaks were summarised using featureCounts in the resting and activated CD4 and CD8 samples. To avoid inflation of expression estimates, the library size for each sample was set to the total number of reads aligned to the genome for that sample. All regions that failed to achieve a CPM greater than 0.5 in at least 2 samples were considered to be unexpressed and were therefore filtered from the analysis. The TMM method was then applied to normalize compositional differences between libraries and the data transformed to log2-CPM with precision weights using voom. Differential expression of the regions was then evaluated between the activated and naïve CD4^+^ and CD8^+^ T cell samples using linear models and robust empirical Bayes moderated t-statistics. P-values were adjusted to control the FDR below 5% using the Benjamini and Hochberg method.

### ATAC-seq data pre-processing and peak calling

75 bp ATAC-seq reads were aligned to the human genome assembly (hg38) using Bowti2 v2.2.5 (bowtie2 -p 4 -X 2000)^48^. For each sample, mitochondrial reads, ummapped reads and low mappability (< 30) reads were filtered out using Samtools (v1.6) function “view”^49^. After filtering, we had a median of 80 million (MAD+/− 13 million) reads per sample. Filtered ATAC-seq reads from resting and activated CD4^+^ and CD8^+^ T cell as well as B cells from two donors were merged using samtools function merge, and peaks were called on the merged bam file using MACS2 (v2.1.0)-callpeak (with parameters –nomodel, –extsize 200, and –shift 100)^50^, such that there were 113,689 peaks after excluding peaks mapping outside the main chromosome contigs. ATAC-seq reads overlapping the peaks were summarized using featureCounts^42^. Peaks in blacklisted genomic regions as defined by ENCODE for hg38 were removed.

### Differential accessibility analysis

Differential accessibility analysis was undertaken using the edgeR v3.20.9^16^ and limma v3.34.9^17^ software packages. The TMM method was applied to normalize compositional differences between libraries^45^. A mean-dependent trend was fitted to the negative binomial dispersions with the estimateDisp function and, and differential accessibility between all cell types was assessed using the quasi-likelihood (QL) framework in edgeR^51,52^, which assesses statistical significance relative to biological variation between the replicate libraries. As is the differential expression analysis, linear models incorporated a correction for a donor batch effect. P-values were adjusted for multiple testing using the Benjamini–Hochberg method. Peaks with a FDR below 5% were defined as differentially accessible regions. Barcode enrichment plots were drawn with limma's barcodeplot function and enrichment p-values were obtained using the fry gene set testing function in edgeR^53^.

For comparison of our ATAC-seq data with the previously published 24 h human T cell activation accessibility signature (GSE118189)^12^, raw data were pre-processed as above and only peaks common to both datasets were considered using the bedtools intersect function (version 2.19.1)^54^. ATAC-seq reads overlapping the intersecting list of peaks (76,812 peaks) were summarized again using featureCounts.

### Enrichment of transcription factor binding motifs

The Homer suite v4.10^55^ was used to determine transcription factor enrichment within ATAC peaks, using the findMotifsGenome.pl function (with parameters hg38 and –size given).

### Annotation of ATAC peaks

Peaks were annotated as 5′ UTR, 3′ UTR, promoter-TSS, exonic, intronic, TTS, non-coding or intergenic using the Homer suite annotatePeaks.pl function and the default setting. Chromatin state(s) of the DA peaks were annotated using the ChIP-seq-defined ChromHMM states from the Roadmap Epigenomics Project and following the method in Corces et al.^56^ In brief, 15 state models were downloaded from the chromatin state learning site for the ‘Primary T helper naïve cells from peripheral blood’ (E038) and ‘Primary T CD8^+^ naïve cells from peripheral blood’ (E047) (https://egg2.wustl.edu/roadmap/web_portal/chr_state_learning.html). We then identified the regions of each ChromHMM state that were overlapped by an DA peak. To determine the significance of these overlaps for each ChromHMM state, we compared the total length of DA peaks covered by the given ChromHMM state to the expected background determined by the total length of the universe of ATAC-seq peaks (13,688 peaks) covered by the ChromHMM state using a binomial test in R.

### Enrichment for chromatin states and gene annotation

Enrichment of ATAC-seq peaks for chromatin states as well as for gene annotations was calculated using GAT v1.3.4^27^. The significance threshold was set up at FDR below 5%.

### Prediction of transcriptional target genes

To identify potential targeted genes for the activation associated accessibility changes we performed enrichment analysis of gene annotations in the proximity of the DA peaks using Genomic Regions Enrichment of Annotations Tool (GREAT)^32^ against the whole genome as background. GREAT links genomic regions with genes by defining a ‘regulatory domain’ for each gene in the genome. Gene regulatory domains were defined with the “Basal plus extension” association rules (proximal 5 kb upstream and 1 kb downstream from the TSS, plus distal extended to the nearest gene’s basal domain but not more than 500 kb). Significantly enriched gene sets were then selected by FDR < 0.05 for binomial tests to identify the regulatory domains with the densest clusters of activation-associated DA peaks and classify them as potential transcription target genes.

### Enrichment of GWAS loci

GREGOR v1.4.0^57^ was used for enrichment analysis of disease-trait associated SNPs in the ATAC-seq peaks. GREGOR calculates enrichment relative to MAF, TSS-distance and number of LD neighbor-matched null SNP sets using the GREGOR parameters: r^2^ threshold = 0.8, LD window size = 1 Mb and minimum neighbor number = 500. For the GWAS SNPs, we created an updated version of Supplementary Table S2 in Maurano et al.^29^. The GWAS SNP set used for analysis was derived from the NHGRI GWAS Catalog, downloaded on August 2, 2017. The catalog contained 41,304 entries at the time of download. We excluded SNPs mapping outside the main chromosome contigs, including the "random" chromosome fragments, SNPs without coordinates in the GRCh37/hg19 human genome assembly. There were 40,929 unique SNP disease/trait combinations that represented 34,421 unique SNP IDs (Supplementary Table S2). Of these, 19,075 were in non-coding regions. Coding regions were defined by the CCDS Project (downloaded from the UCSC genome browser at http://hgdownload.cse.ucsc.edu/goldenPath/hg38/database/ccdsGene.txt.gz on August 4, 2017). As in Maurano et al.^29^, we also grouped SNPs into classes of similar diseases or traits but some of the categories were updated so they could better reflect the new and extended list of SNPs. Categories comprised: aging related; immune system; cancer; cardiovascular diseases and traits; metabolic disorder; drug metabolism; hematological parameters; kidney, lung, or liver; miscellaneous; serum metabolites; neurological/behavioral; neurological/autoimmune; parasitic or bacterial disease; quantitative traits; radiographic (primarily bone density); viral disease.

### Nucleosome occupancy

Nucleosome occupancy was defined with the “occ.bedgraph” files using the NucleoATAC v0.3.2.1 package^58^.

### In situ Hi-C data pre-processing

Each sample was aligned to the hg38 genome using the presplit_map.py script in the diffHic package v1.10.0^33^. Data were pre-processed and artefacts removed as per Johanson et al.^59^.

### Differential interaction (DI) analysis

DIs between all five libraries were detected using the diffHic package^33^ at two different resolutions, 100 kbp and 25 kbp. Read pairs were counted into 25 or 100 kbp bin pairs (with bin boundaries rounded up to the nearest MboI restriction site) using the squareCounts function. This yielded a matrix of read pair counts for each bin pair in each library. All bins with counts less than 5 were discarded along with bins on the sex chromosomes. Bins containing blacklisted genomic regions as defined by ENCODE for hg38^60^ were also removed. Filtering of bin-pairs was performed using the filterDirect function, where bin pairs were only retained if they had average interaction intensities more than sixfold higher than the background ligation frequency. The ligation frequency was estimated from the inter-chromosomal bin pairs from a 2 Mbp bin-pair count matrix. Bins on the first diagonal of the interaction space are also removed with the filterDiag function.

For the retained bin pairs, counts were normalized between libraries using a LOESS-based approach to account for abundance-dependent biases. This was performed using the normOffsets function to obtain a matrix of offsets with bin pairs less than 100 kbp (for the 25 kbp) or 150 kbp (for the 100 kbp) from the diagonal normalized separately from other bin pairs. Tests for differential interactions were performed using the quasi-likelihood (QL) framework^51,52^ of the edgeR package (v3.20.9). The design matrix was constructed using a layout that specified the cell lineage to which each library belonged and which individual the cells were sampled from. Using the counts and offsets for all bin pairs, a mean-dependent trend was fitted to the negative binomial dispersions with the estimateDisp function. A generalized linear model (GLM) was fitted to the counts for each bin pair^16^, and the QL dispersion was estimated from the GLM deviance with the glmQLFit function. The QL dispersions were then squeezed toward a second mean-dependent trend, using a robust empirical Bayes strategy^47^ to share information between bin pairs. A P-value was computed for each bin pair using the QL F-test, representing the evidence against the null hypothesis, i.e., no difference in counts between groups. P-values were adjusted for multiple testing using the Benjamini–Hochberg method. If a bin pair had a FDR below 5%, it was defined as a DI. To reduce redundancy in the results, DIs adjacent in the interaction space were aggregated into clusters using the diClusters function to produce clustered Dis. DIs were merged into a cluster if they overlapped in the interaction space, to a maximum cluster size of 500 kbp (for the 25 kbp) or 1Mbp (for the 100 kbp) to mitigate chaining effects. The significance threshold for each bin pair was defined such that the cluster-level FDR was controlled at 5%. Cluster statistics were computed using the combineTests and getBestTest functions from the csaw package v1.12.0^61^. Clustered DIs were used to report the number of differences between the libraries. The 25 kbp unclustered DIs were used for overlap analysis and integration with other data types. Contact matrices were created from the libraries using the inflate function in diffHic for various bin sizes with no filtering. Contact matrices from biological replicates were summed.

### Detection of TADs

TAD breakpoints were detected with the TADbit v0.2.0.5 python based software^34^. Read pairs were counted into 50 kbp bin pairs (with bin boundaries rounded up to the nearest MboI restriction site) using the squareCounts function of diffHic with no filter. This yielded a count matrix containing a read pair count for each bin pair in each library. The count matrix was converted into a contact matrix for each somatic chromosome with the inflate function of the InteractionSet package^61^. Replicate contact matrices were summed to obtain one matrix for each cell type. The TADbit tool *find_tad* was run on the raw counts specifying normalized = FALSE. Only TADs boundaries with a score of 7 or higher were included in the results.

### Detection of differential TAD boundaries (DTBs)

Changes in TAD boundaries between activated and resting cells were assessed with the diffHic and edgeR packages using the approach described in Chapter 8 of the diffHic User's Guide. This approach adapts the statistical strategy recently developed for differential methylation^62^ to identify TAD boundaries that are significantly strengthened or weakened between cell types. The strength of each TAD boundary was assessed based on the upstream vs downstream intensity contrast at that genomic loci, defined as the ratio of upstream to downstream Hi-C read pairs anchored at that genomic region. edgeR was used to test whether the ratio increased or decreased for each boundary upon T cell activation. This method directly assesses differential boundary strength relative to biological variation without needing to make TADs calls in individual samples. Upstream and downstream read counts were determined for the same 50 kbp genomic regions as used for the TADbit analysis. An upstream (downstream) read was defined as one with anchors in the 50 kbp region and 0–1 Mbp upstream (downstream) of the region. Low abundance regions with average log2-counts per million below 0.8 were removed. An appropriate edgeR linear model was fitted to the counts for all the T cell samples. Tests for differential TAD boundaries were performed using the QL framework of the edgeR package as described in the DI analysis section above. Regions with FDR below 0.05 were considered to be significantly different.

### Detection of A/B compartments

A/B compartments were identified at a resolution of 50 kbp using the method outlined by Lieberman-Aiden et al.^63^ using the HOMER HiC pipeline^55^.

After processing with the diffHic pipeline libraries were converted to the HiC summary format using R. Then input tag directions were created for each library with the makeTagDirectory function specifying the genome (hg38) and restriction enzyme cute site (GATC). Biological replicates tag directories for each cell type were summed. The runHiCpca.pl function was used on each library with -res 50,000 and specifying the genome (hg38) to perform PCA to identify compartments. To identify changes in A/B compartments between libraries, the getHiCcorrDiff.pl function was used to directly calculate the difference in correlation profiles. We identified regions with an A-to-B or B-to-A compartment flip that showed a correlation profile lower than 0 and regarded them as differential compartments.

### Visualization of the data and plots

Plots were performed using R and ggplot. Multi-dimensional scaling (MDS) plots were constructed using the plotMDS function in the limma v3.34.9 package^17^ applied to the filtered and normalized log2-counts-per-million values for each library. The removeBatchEffect function of the limma package was used to correct for effect of the individual and batch in the data. Plaid plots were constructed using the contact matrices and the plotHic function from the Sushi v1.22.0 R package^64^. The inferno color palette from the viridis v0.5.1 package^65^ was used and the range of color intensities in each plot was scaled according to the library size of the sample, to facilitate comparisons between plots from different samples. DI arcs were plotted with the plotBedpe function of the Sushi package. The z-score shown on the vertical access was calculated as − log10 (P-value). RNA-seq coverage was plotted with the plotBedgraph function of the Sushi package and Integrative Genomics Viewer, IGV. UpSet plots were generated using the UpSet function in the ComplexHeatmap package in R^66^.

### Data intersection

Intersection between pairs of the genomic regions was performed using bedtools intersect (v2.19.1)^54^.

## Supplementary Information

Supplementary Figure S1.

Supplementary Figure S2.

Supplementary Figure S3.

Supplementary Figure S4.

Supplementary Figure S5.

Supplementary Figure S6.

Supplementary Figure S7.

Supplementary Table S1.

Supplementary Table S2.

Supplementary Table S3A.

Supplementary Table S3B.

## Data Availability

Raw and processed Hi-C, ATAC-seq, and RNA-seq data from this study are available from the NCBI Gene Expression Omnibus as series GSE126117 (reviewer token yxupgogepbsvrqp).
