# Supplementary material for: Multi-level remodelling of chromatin underlying activation of human T cells
Source: Sci Rep. 2021 Jan 12;11:528. doi: 10.1038/s41598-020-80165-9 (PMC7804404; doi:10.1038/s41598-020-80165-9)
Supplement: Supplementary file 1 — Supplementary Figure S1. [file 41598_2020_80165_MOESM1_ESM.pdf]

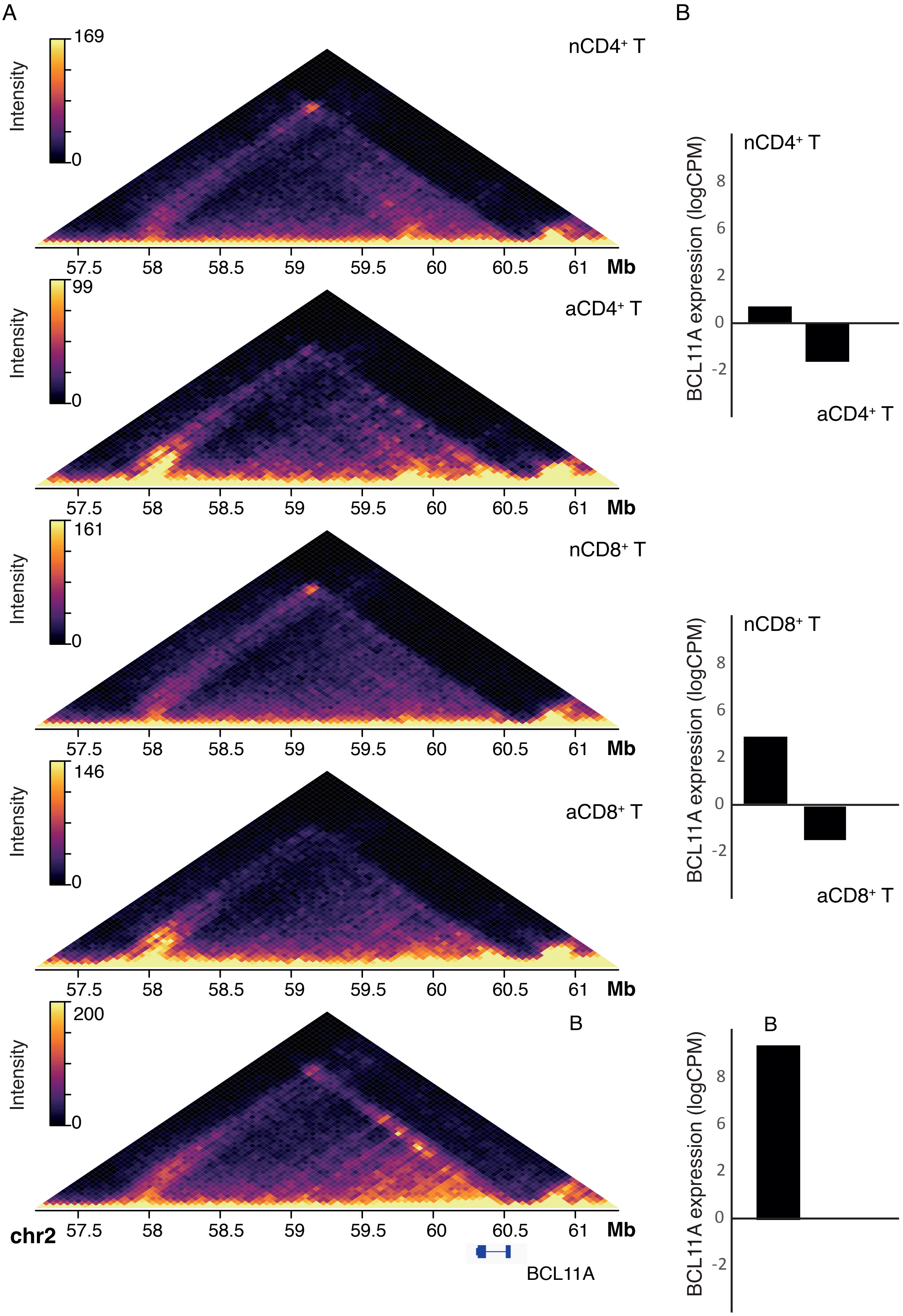

Figure S1. Chromatin structure and gene expression are immune cell type-specific (A) In-situ Hi-C contact matrices for a 2.5 Mb region on chromosome 2 that includes the BCL11A locus at 50 kbp resolution. The top four Hi-C matrices display data from resting and activated T cells with minimal genome organisation at the gene, while the bottom Hi-C matrix from B cells shows distinct genome organisation at the BCL11A locus. Color scale indicates number of reads per bin pair. (B) Bar plots showing the expression of BCL11A across resting and activated $CD4^+$ and $CD8^+$ T cells, and B cells.
