# Supplementary material for: Multi-level remodelling of chromatin underlying activation of human T cells
Source: Sci Rep. 2021 Jan 12;11:528. doi: 10.1038/s41598-020-80165-9 (PMC7804404; doi:10.1038/s41598-020-80165-9)
Supplement: Supplementary file 2 — Supplementary Figure S2. [file 41598_2020_80165_MOESM2_ESM.pdf]

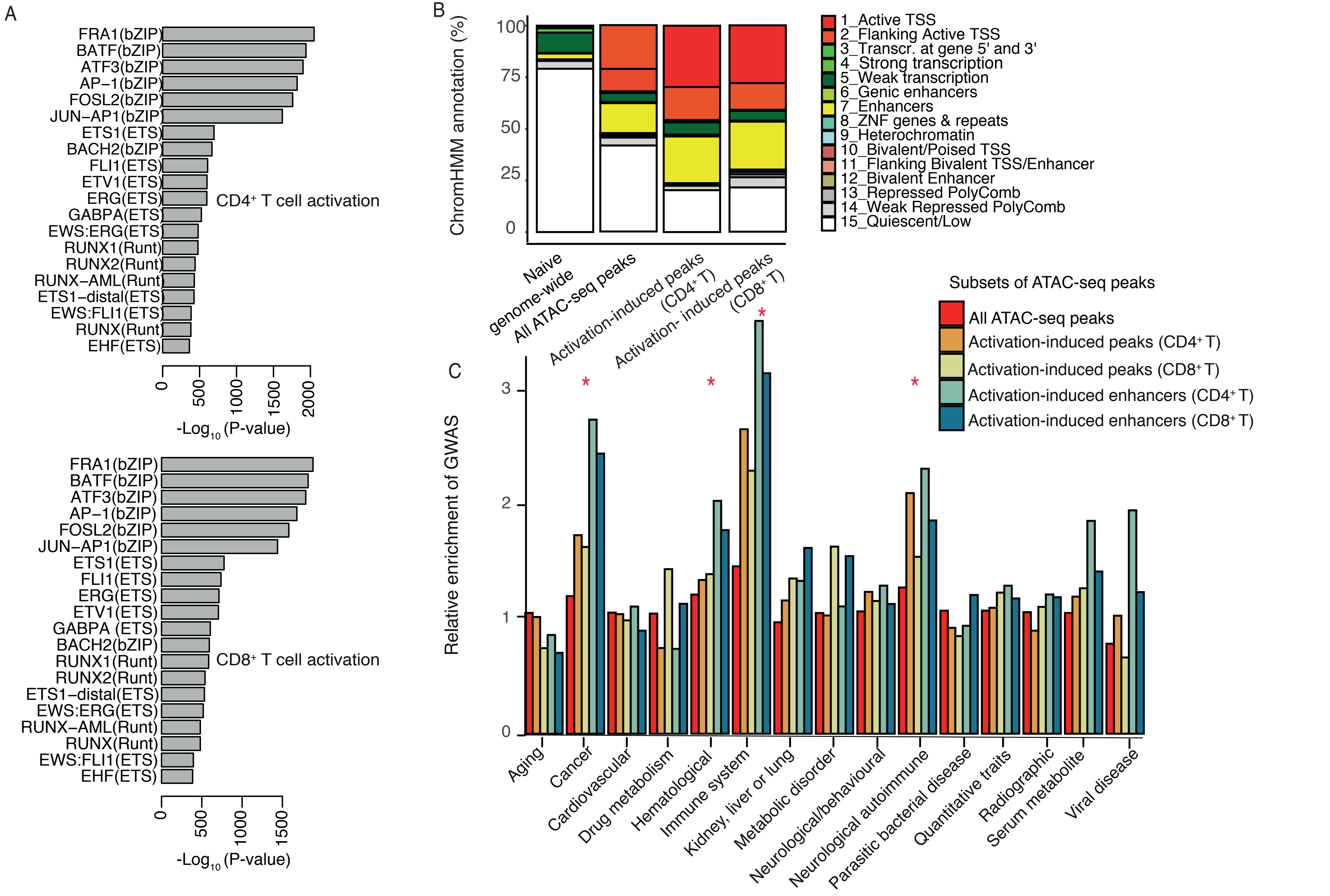

Figure S2. T cell activation-induced accessibility changes are enriched for enhancers and other regulatory elements and GWAS SNPs. (A) Bar plots showing the top-ranked TFBS motifs that are enriched in activation-induced accessibility changes in CD4+ and CD8+ T cells, where the X-axis represent the significance of the enrichment (−log10 P-value, cumulative binomial test), and Y-axis each of top-ranked motifs. (B) Bar plots showing the distribution of the predicted chromatin states (ChromHMM) in subsets of peaks. (C) Bar plots showing enrichment of GWAS SNPs in subsets of ATAC-seq peaks. Shown are the relative enrichment of GWAS (observed/ expected number of overlapping SNPs) (Y axis) for each SNP category (X axis). Red asterisks denote SNP categories for which subsets of ATAC-seq peaks showed significant enrichment by GREGOR.
