# Supplementary material for: Multi-level remodelling of chromatin underlying activation of human T cells
Source: Sci Rep. 2021 Jan 12;11:528. doi: 10.1038/s41598-020-80165-9 (PMC7804404; doi:10.1038/s41598-020-80165-9)
Supplement: Supplementary file 3 — Supplementary Figure S3. [file 41598_2020_80165_MOESM3_ESM.pdf]

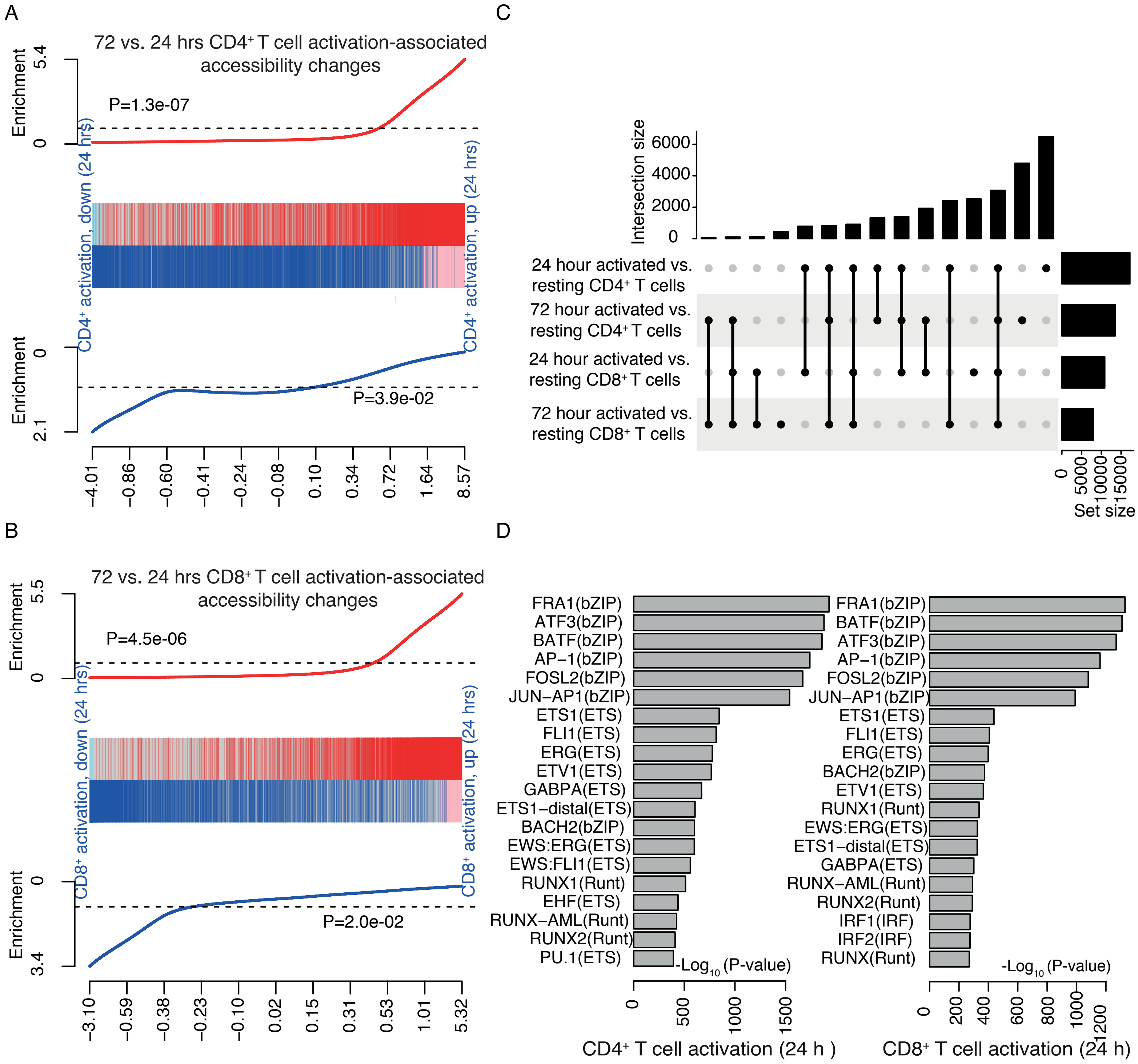

Figure S3. T cell activation induces extensive changes in chromatin accessibility (A) Barcode enrichment plot showing that DA peaks in CD4+ T cells activated for 72 hours are similarly ranked in CD4+ T cells activated for 24 hours. Peaks are ordered from left to right on the plot from most down-regulated to most up-regulated upon 24 hours of activation, forming a shaded middle horizontal bar, with log2-fold-changes shown by the x-axis. DA peaks significantly up-or down-regulated after 24 hours of activation (FDR <0.05) are marked by red and blue vertical bars, respectively. The red enrichment worm at the top of the plot shows the local density of red vertical bars while the blue enrichment worm at the bottom of the plot shows local density of the blue vertical bars. P-values show significance of the enrichments (fry gene set tests). The enrichment pattern shows strong concordance of the 24- and 72- hour activation-associated changes in CD4+ T cells. (B) Barcode enrichment plot showing that DA peaks in CD8+ T cell activated for 72 hours are similarly ranked in CD8+ T cell activated for 24 hours. (C) UpSet plots showing the number of DA peaks from each comparison (24 hour activated vs. resting CD4+ T cells, 72 hour activated vs. resting CD4+ T cells, 24 hour activated vs. resting CD8+ T cells, 72 hour activated vs. resting CD8+ T cells) and their intersections. Horizontal bars illustrate the total number of DA (set size) from each comparison. Vertical bars show the number of common DA peaks (intersection size) from a given set of comparisons (filled connected circles). (D) Bar plots showing the top-ranked TFBS motifs that are enriched in the 24 hour activation-induced accessibility changes in CD4+ and CD8+ T cells, where the X-axis represent the significance of the enrichment (−log10 P-value, cumulative binomial test), and Y-axis each of top-ranked motifs.
