# Supplementary material for: Multi-level remodelling of chromatin underlying activation of human T cells
Source: Sci Rep. 2021 Jan 12;11:528. doi: 10.1038/s41598-020-80165-9 (PMC7804404; doi:10.1038/s41598-020-80165-9)
Supplement: Supplementary file 4 — Supplementary Figure S4. [file 41598_2020_80165_MOESM4_ESM.pdf]

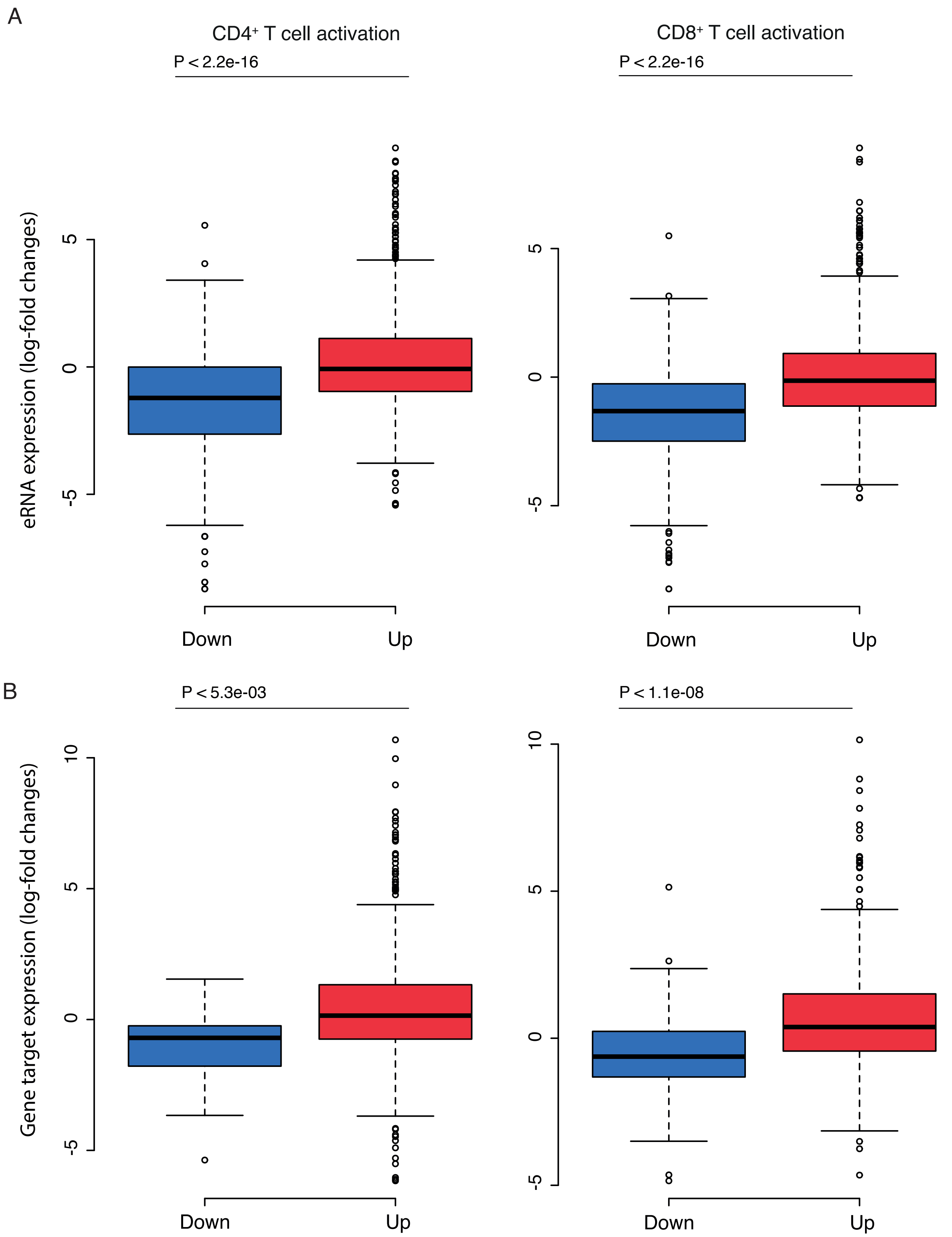

Figure S4. Variation of chromatin accessibility at the activation-induced peaks is associated with changes in expression of eRNAs and targets genes. Distribution of the expression of (A) eRNAs and (B) predicted enhancer target genes across the activation-induced peaks that gained and lost accessibility is represented as box plots (central bar represents the median with boxes indicating interquartile range, values of the upper and lower quartiles are represented in with whiskers and outliers with circles). Statistical comparisons were made with the unpaired Wilcoxon test.
