# Supplementary figures and images for: Multi-level remodelling of chromatin underlying activation of human T cells
Source: Sci Rep. 2021 Jan 12;11:528. doi: 10.1038/s41598-020-80165-9 (PMC7804404; doi:10.1038/s41598-020-80165-9)

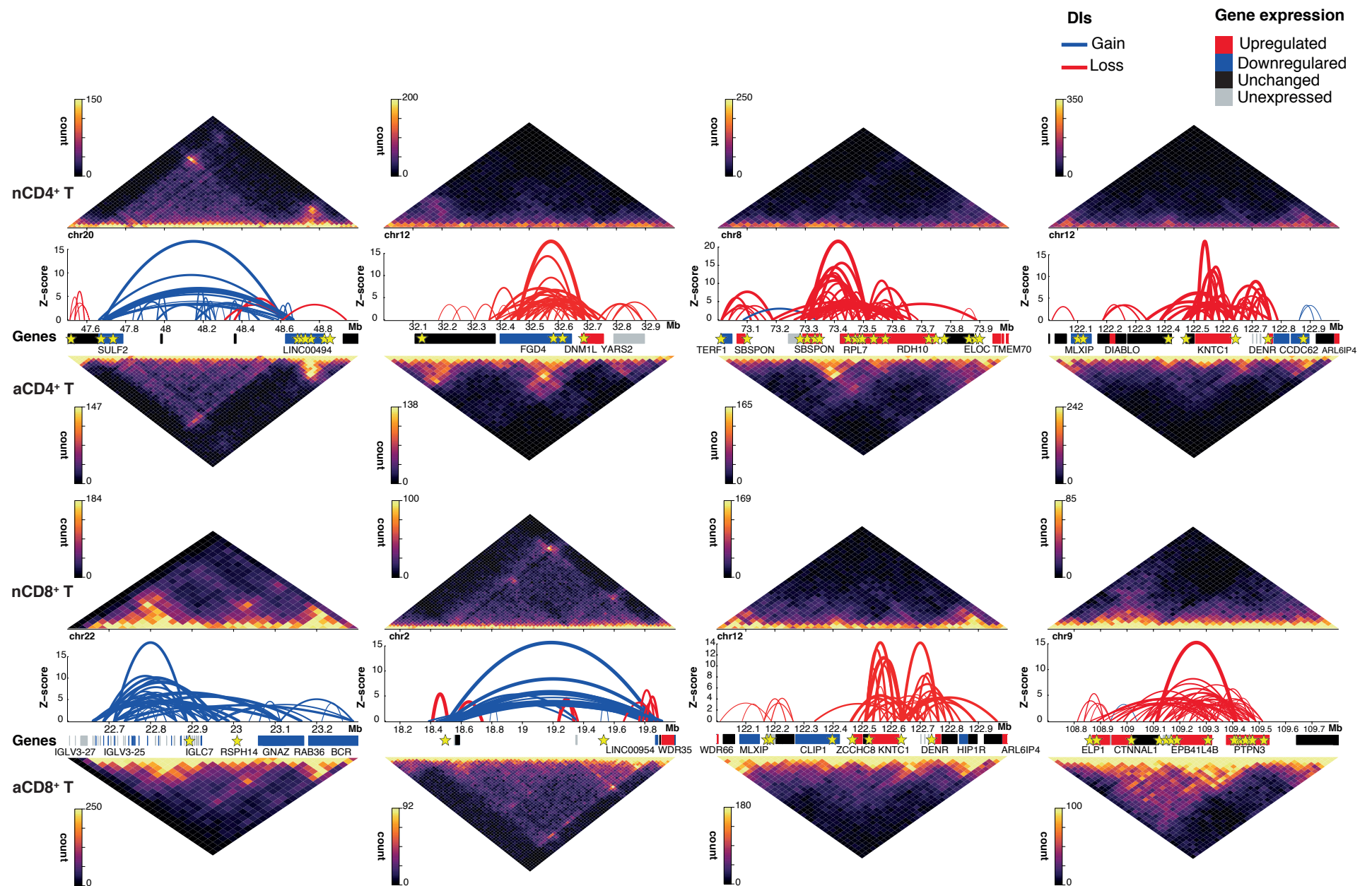

Supplement: Supplementary file 5 — Supplementary Figure S5. [file 41598_2020_80165_MOESM5_ESM.pdf]
