# Supplementary material for: Multi-level remodelling of chromatin underlying activation of human T cells
Source: Sci Rep. 2021 Jan 12;11:528. doi: 10.1038/s41598-020-80165-9 (PMC7804404; doi:10.1038/s41598-020-80165-9)
Supplement: Supplementary file 6 — Supplementary Figure S6. [file 41598_2020_80165_MOESM6_ESM.pdf]

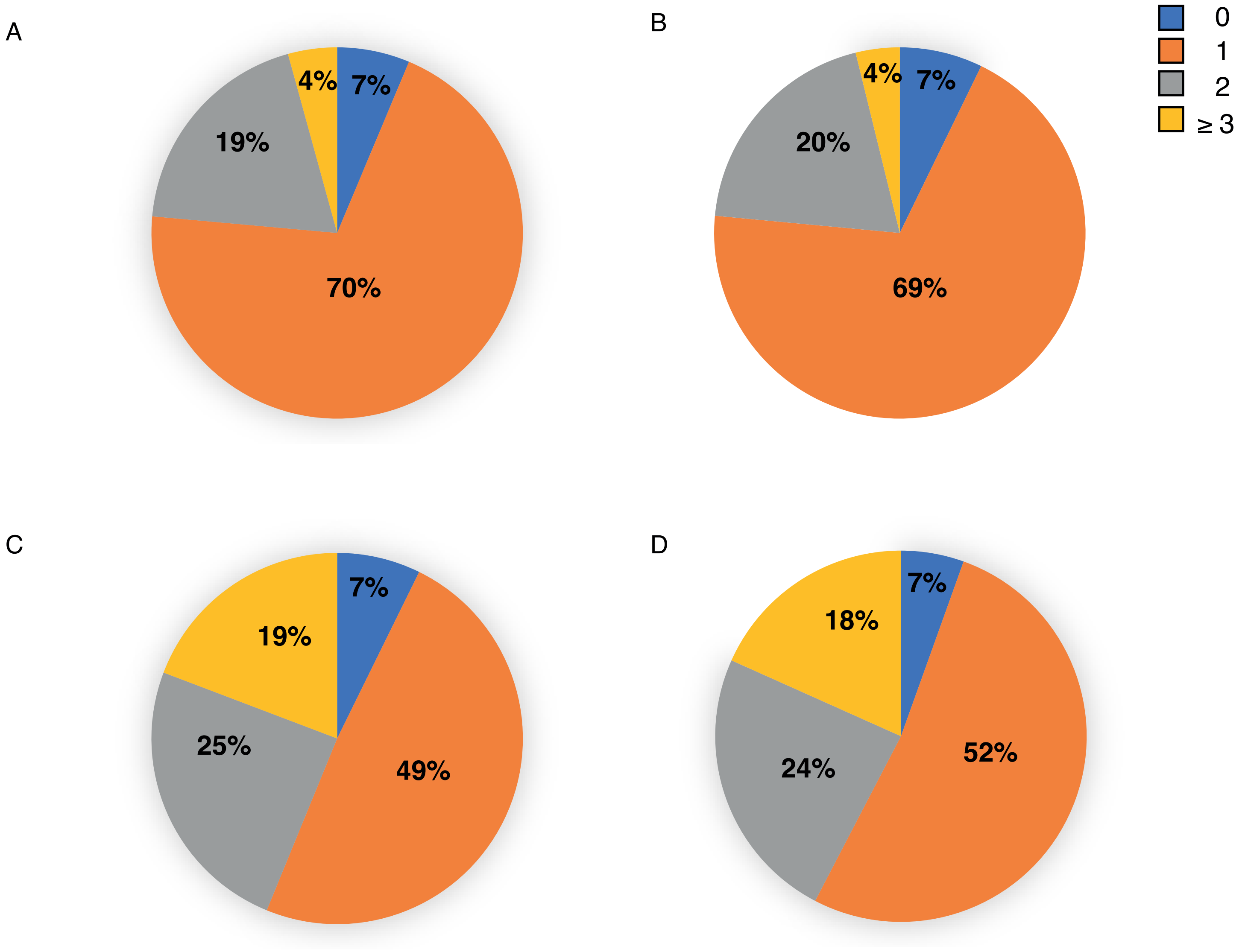

Figure S6. T cell activation results in partitioning of genome topology. Pie chart representation of the distribution of (A) TADs in resting CD4$^+$ T cells overlapping 0,1, 2 or ≥ 3 TADs in resting CD8$^+$ T cells, (B) TADs in resting CD8$^+$ T cells overlapping 0,1, 2 or ≥ 3 TADs in resting CD4$^+$ T cells (C) TADs in resting CD4$^+$ T cells overlapping 0,1, 2 or ≥ 3 TADs in activated CD4+ T cells, and (D) TADs in resting CD8$^+$ T cells overlapping 0,1, 2 or ≥ 3 TADs in activated CD8$^+$ T cells.
