# Supplementary material for: Multi-level remodelling of chromatin underlying activation of human T cells
Source: Sci Rep. 2021 Jan 12;11:528. doi: 10.1038/s41598-020-80165-9 (PMC7804404; doi:10.1038/s41598-020-80165-9)
Supplement: Supplementary file 7 — Supplementary Figure S7. [file 41598_2020_80165_MOESM7_ESM.pdf]

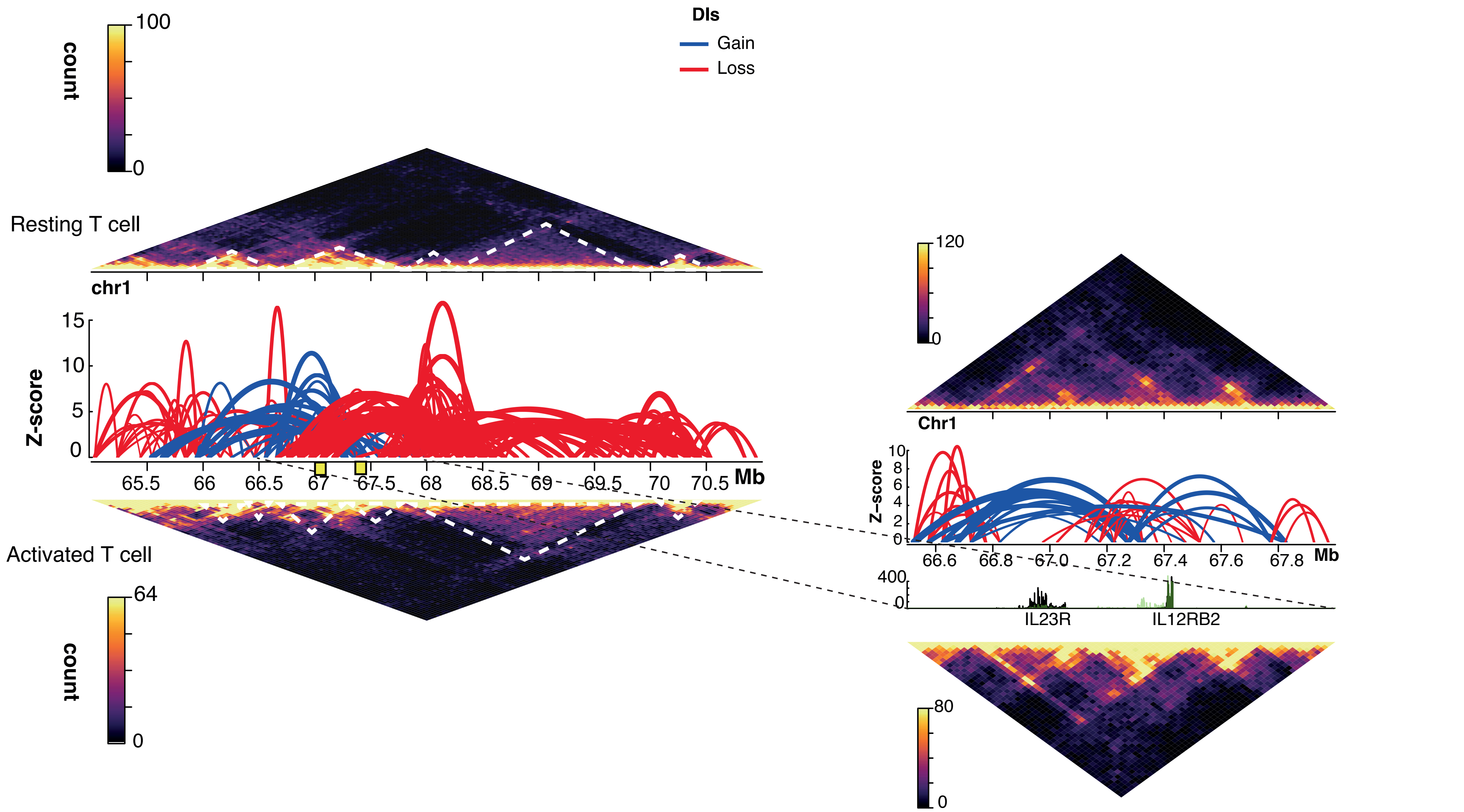

Figure S7. T cell activation results in partitioning of genome topology. In-situ Hi-C contact matrices plotted at 50 kbp in resting and activated T cells over genes that have been associated with T cell activation such as IL23R and IL12RB2. Colour scale of contact matrix indicates number of reads per bin pair. Locations of the TADs are marked by white dotted lines as called by TADbit. Significant differential interactions (DIs) as determined by the diffHic pipeline at 100 kbp (FDR<0.05) are represented by arcs in the centre where the vertical axis is the z-score (-log10 p-value as calculated by edgeR). Each arc connects the two interacting bins and therefore identifies the relevant bin pair. Red and blue arcs represent strengthened (logFC>0) and weakened (logFC<0) DIs, respectively, in response to activation. The zoomed inset regions show in-situ Hi-C contact matrices, significant differential interactions at 25 kbp resolution (FDR<0.05) and RNA sequencing coverage plots for the IL23R and IL12RB2 loci. Light and dark green coverage plots represent activated and resting T cells, respectively.
